# Monthly Summary

**Published:** 1895-01

**Authors:** 


### Monthly Summary.

ABRASIONS OF THE SKIN.—Tinct. benzoin comp. is of especial value, according to Dr. J. L. G. Sherill (*Amer. Therapist*), in abrasions of the skin, in conditions such as chapped hands, lips, fissured nipples, etc. It is also of service in eczema where there is a tendency to cracking. The peculiar benefit to be derived from its use in such conditions lies in the fact that after evaporation the gum forms a thin coating over the abraded surface, in this way protecting it from external influences and also from infection. The surgical use of compound tincture benzoin should be more widely extended. In case of injury about the hand—whether it be slight incision in which suture would not be necessary, or whether it be excessive laceration and contusion, and especially injuries occurring in the use of machinery—he has found the application of compound tincture benzoin to be especially useful. The manner in which it should be used is as follows: After carefully cleansing the wound, removing all foreign substances, irrigating the wound with an antiseptic solution if there is any chance or indication of infection, and thoroughly checking all hemorrhage a layer of surgical cotton is placed around the wound. If the injury is about the hand the fingers should, of course, be separated and kept apart by layers of cotton. After cotton is applied the compound tincture benzoin is poured down next the surface of the wound, saturating the cotton immediately surrounding the injured tissues. This drug, after undergoing evaporation, will form a coating with the cotton which will hermetically seal the part, thereby rendering it perfectly aseptic. Benzoin in itself is an antiseptic, although of inconsiderable strength. There will be some slight smarting upon the application of this tincture, which, however, will not seriously inconvenience the patient, and which is due entirely to the fact that it is carried by an alcoholic medium.—*Medical and Surgical Reporter.*

---

HOW METALS GET TIRED.—Fatigue of metals is an expression which has come into use only in recent years, and it describes a condition of the material not previously understood. The expression stands for a straining of the relationship to each other of the molecules of which the metal is constituted.

In the metals, there is a point in their resistance to pulling, bending, or crushing, known as the elastic limit, the point at which permanent strain commences. The elastic limit of iron or mild steel, for example, in its normal condition, is reached roughly speaking, when about half the breaking load is applied. If the stresses brought to bear on a piece of metal are within this limit, it will sustain these stresses without injury, however long they may be applied. If the stresses exceed the elastic limit, in however small a degree, fatigue of the metal will result, and if they are continued, breakage will inevitably take place. To guard against accident from such insidious fatigue, it has now become usual, in the best practice, to withdraw permanently from use parts of machinery, such as railway crank and carriage axles, on the soundness of which the safety of many lives may depend, after they have performed a certain number of revolutions, even though no flaw or sign of injury can be detected.

If, however, metals are strained beyond the elastic limit, but not broken, and if the straining is not continued, the material will recover its elasticity by rest alone. Some years ago Prof. B. W. Kennedy demonstrated by many experiments the recuperative property of metals after fatigue.

Bars of iron and steel, strained in a testing-machine beyond the elastic limit, and so weakened thereby that if they were tested again the following day they would take permanent set at one-third or less of their former load, would, if allowed to rest for about two years, be found not only to have recovered their original elastic limit of strength, but to have exceeded it, and to have become stronger than before in the direction in which they had been pulled. If the period of rest was materially shortened, the restoration of strength was found to be incomplete.—*Knowledge.*

---

THE CAUSE OF DEATH FROM CHLOROFORM.—Heart weakness is so generally assumed to be the first warning of danger in chloroform narcosis that during the anæsthesia the pulse is more closely watched than the breathing. That chloroform has no direct action to the heart, however, and that it kills by inducing respiratory paralysis, is the conclusion of Surgeon Lieutenant-Colonel Lawrie, as reported in the *British Medical Journal* for July 7th. At a recent meeting of the Royal Medical and Chirurgical Society he contended that the experiments, including those performed under the auspices of the Hyderabad Commission, proved that death from chloroform was due to respiratory failure, and that the practical point to remember during its administration was that the condition of the pulse was quite subsidiary, but that the state of the respiration should be closely watched. Chloroform being an irritant, protoplasm is irritated and destroyed by either its liquid or its vapor. When it is injected into the substance of a muscle, such as the heart or the biceps, motion is arrested in the same manner as it is by hydrochloric acid or any other irritant. He argued that in poisoning from the inhalation of chloroform this irritant action could no more take place in the heart than in the biceps, and hence might be ignored in considering the clinical question of accidental death under this anæsthetic. In his experiments with animals, chloroformed blood sent to the heart alone produced no effect whatever, but when it was sent to the brain alone the narcotic acting on the brain centers produced its usual effects. From tracings of the pulse and of the breathing, he demonstrated that chloroform anæsthesia without respiratory complication was free from risk.

From much experience with chloroform, Mr. Horsley also was convinced that it was the arrest of respiration which resulted in death, and that in the majority of cases of danger inversion of the patient and artificial respiration would cause recovery. Mr. Gaskell and Mr. Shore agreed that respiration failed first, but held that chloroform had a direct action on the heart also. Dr. Lauder Brunton's experience was that chloroform always paralyzed the respiratory center before enough had been taken to paralyze the heart. A number of the accidental deaths were due, not to the chloroform, but to the operation itself, to asphyxia, or to noxious substances circulating in the blood. Mr. Lawrie concluded by stating that he had found it impossible to teach careless men to administer chloroform safely, and that heart failure might be indirectly produced by stimulation of the vagus through irregular breathing. He had noticed no difference in the effects of chloroform on different races or nationalities. In seven hundred cases of chloroform narcosis the pulse had been carefully watched, but it had given no reliable indications of danger.—"Med. and Surg. Reporter."

---

A CAUSE FOR BALDNESS.—Dyspepsia is not only one of the most common diseases, but it is also one of the most common causes for the loss of hair. Nature is very careful to guard and protect and supply the vital organs with the proper amount of nutriment, but when she cannot command a sufficient quantity of blood supply for all the organs she very naturally cuts off the supply of parts the least vital, like the hair and nails, so that the most important organs, like the heart, lungs, etc., may be better nourished and perform their work more satisfactorily. In cases of severe fevers one can readily see how nature economizes. If one will examine a hair very closely from the beard or head it will be seen that it gives somewhat of a history of an individual during the time it was growing. It will be observed that it shows attenuated places, showing that at some period of its growth the blood supply was deficient from overwork, anxiety or underfeeding. Speaking of dyspepsia being one of the most common causes of alopecia, I will add that a very common cause of indigestion is irregularity of meal hours. The human system seems to form habits, and it performs its functions to a great measure in accordance with the habits formed. This seems to be particularly so in regard to eating, and you might say drinking. Your stomach gets into the habit of accepting your meals at a certain hour every day, and at that hour it is ready for it. If you, however, take meals at irregular hours you take your stomach by surprise, and it does not know when to expect a meal, and it is not in that state of readiness for prompt and perfect performance of its work. Be more careful about what you eat, when you eat it, and you will have less dyspepsia and fewer bald heads.—*Ed. Charlotte Med. Jour.*

---

I have used the following mode of root filling for three years, but consider it most satisfactory and valuable where we are unable to remove the entire contents:

Having adjusted the rubber-dam I remove all the contents from pulp-chamber and canals possible. I do not think the sensation sometimes felt in these roots is from air pressing on the nerve filaments, but from a small portion of the remaining nerve. I take oil of eucalyptus and enough aristol to color the oil to a dark brown, and with a few threads of cotton wound around the broach, I pump sufficient into canal to thoroughly saturate it. Then proceed to fill in the ordinary way with chlora-percha and gutta-percha points.

I, of course, take all pains possible to remove all the pulp and fill the chamber as thoroughly as possible. But in many instances I know a part is left, yet have had no trouble.—*J. F. Wallace, in Items of Interest.*

MEDICAL ANTISEPSIS.—M. Petresco, of Bucharest, (*Gaz. des. Hop.*), reports results which he has obtained by medical antisepsis which he has largely used as a therapeutic measure. He has employed nearly all the antiseptics in use. His conclusions are as follows:

1. A local medical or general antisepsis at a distance on the histological elements and on the pathogenic germs or on their toxines can be obtained which is analogous to local surgical antisepsis.

2. There are several chemical substances (fixed or volatile) which upon absorption can, even in remote parts, kill or attenuate certain pathogenetic micro-organisms.

3. There are some medicaments which neutralize or modify chemistry the toxines of pathogenic germs.

4. The best therapeutic agents to produce medical antisepsis are the products, more or less condensed, of the aromatic series, such as the essences. The essences are the antiseptics *par excellence* as much in medicine as in surgery. It is by the essences or their vapors that impregnation is obtained, a modification, more or less profound, of the organism, and in consequence a medical immunity. There is created a medical sterilization of the inferior tissues analogous to the sterilization *in vitro* in a culture. By this sterilization or antisepsis of the interior microbe or ptomaine, auto-intoxication is prevented in sufferers from contagious diseases.

5. Medical antisepsis should be utilized in nearly all internal diseases of a microbic nature, as it has been utilized in the external parasitic diseases.

6. To establish the basis of medical antisepsis we must have a therapeusis which will act as a microbocide or destroy toxines or ptomaines.—*Med. and Surg. Reporter.*

---

COLD WATER AS AN ANTIPYRETIC.—Dr. Fisher has arrived at the following conclusions:

1. That cold water is the best antipyretic used to-day.

2. That it is easily obtainable and is therefore adapted to all classes of cases, both rich and poor.

3. The mode of application is so simple that it adapts itself to the hospital and equally as well to private practice, and can be applied without any distinct training.

4. Cautiously given it stimulates.

5. Carelessly used and longer than required it depresses and will produce subnormal temperature.

6, That rectal temperature should be taken and the bath at once discontinued when temperature falls to 101°, as it will then continue to fall.

7. That a stimulant administered before the bath may be necessary and should be given where there is a feeble heart.

8. That the temperature indicates when to commence and when also to discontinue the hydropathic treatment.

9. Unnecessary blanketing after a bath is injurious and will produce copious perspiration, which I believe weakens the patient.

10. The temperature of the room should always be between 68° and 72°.—*Post Graduate.*

---

NEW MEANS OF LOCAL ANÆSTHESIA.—One Dr. K. L. Schleich, of Austria, claims to have discovered that absolute immunity from pain, even during pratracted operations, may be obtained by subcutaneous injections of a sugar or salt solution, or of merely cold distilled water; that the results induced are to all intents and purposes identical with those obtained by like employment of cocaine. He adds:

The patient may remain perfectly conscious during the amputation of hand and foot without undergoing the tortures so often inflicted upon the battle-field or the exceptional dangers of syncope ever present in the operating-room when general narcosis is resorted to.

It is declared that this discovery has already borne the test of several experiments and is about to be applied in the hospitals of Vienna. The explanation of the phenomenon is:

Local insensibility to pain is induced in the case of cocaine by purely chemical changes, while cold water and solutions of sugar and salt act mechanically through high pressure and low temperature. Under the influence of high pressure and sudden low temperature the blood and lymph are driven from the places operated upon to where the pressure is less. The tissues are thus deprived of their supply of blood and temporary paralysis of nerves results.

The foregoing seems to have met with favor in numererous quarters, and in one instance at least a surgeon of authority affirms that "its importance is all the more undoubted, seeing that if in a given case cold water alone should fail to produce the needful degree of insensibility, a weak and absolutely harmless solution of cocaine would prove certainly efficacious.

Whether true or false, it is hardly probable that surgeons will give up the use of cocaine, which is deemed certain, in favor of a method which will always be apt to carry with it a feeling of indefiniteness.—*Medical Age.*

---

HYDROTHERAPY IN FRACTURES.—More than twenty years ago the writer adopted the practice of treating fractured limbs for a short time with various applications of water before putting the parts into a permanent dressing. By this means it is found possible to avoid much of the pain, swelling and discomfort which the patient usually suffers during the first few days after the application of the dressing, and to secure more speedy and complete union, with less disability of the overlying muscles and the contiguous joints. We have frequently found hot fomentations most useful in these cases. The application of hot fomentations, or soaking the affected parts in hot water for an hour or two, almost invariably has the effect to relieve pain from circulation in the contused vessels, to prevent swelling, to overcome muscular spasm and rigidity and to promote the recovery of the patient. If there is much displacements of the fragments it is, of course. important that the parts should be drawn as nearly as possible into position. The parts can be retained by a temporary pasteboard splint and light bandage during the application until the permanent dressing is applied. Dr. T. Morton, of Philadelphia, recently reported a case (*New Eng. Med. Monthly*) in which an un-united fracture of the leg was made to unite three or four months after the accident occured by applications of hot water and massage.—*Mod. Med.*

---

UNCOMMON CASE OF SALIVARY CALCULUS.—Salivary calculus deposits at the ends of whartonian and steno ducts are easily diagnosed, through their locality as well as their consistency. The glands are generally considerably swollen, and the form of the deposit is oval. The deposit is found more frequently in the sub-maxillary and less in the parotid. The knowledge of their origin has not been fully brought to light. It is generally accepted to be a precipitation of uric acid salts around a foreign substance. In the majority of cases they will be found at the principal outlet of the glands, close to the buccal opening, as it is easy for a foreign substance to get into the opening of the duct. More difficult is the diagnosis if the deposit is deeper in the duct or even in the gland itself. In the latter it is scarcely probable that any foreign substance could find its way into them. It would be that it was forced in with the mucus. When such is the case, suppuration generally takes place, particularly in the parotid. The treatment is very simple; when there is a tendency to recurrence extirpate the glands.—*Dominion Dental Journal.*

---

WHY PEOPLE BECOME DEAF.—It has taken the medical world a great many years to discover that loss of hearing is almost invariably caused by some disease of the throat or nose or both. But very recent researches in these fields have demonstrated this fact beyond question, and it is now admitted by the more advanced medical men that, aside from rupture of the ear-drum, there is scarcely a symptom of defective hearing which is not traceable directly to the condition of the nose and throat.

In view of the new discoveries, ear specialists are finding their occupation gone, save as they make their particular branch an assistant in further investigation. It is said, as we have already pointed out, that the use of smelling-salts is one of the most prolific causes of deafness, operating by weakening the olfactory nerves, and through them the auditory system. All strong or pungent odors should be avoided as far as possible, especially those which act upon the secretory processes and, as the popular expression goes, "make the nose run."—*Medical Brief.*

---

CHLOROFORM NAUSEA.—With regard to chloroform nausea, too little regard is paid to prophylaxis. In the case of sea-sickness nothing is so important as treatment of the stomach before the voyage; and nothing will prevent chloroform sickness like careful dieting and some compound rhubarb powder during the three days preceding operation. In emergency cases washing out the stomach before operation would be most useful. To treat the nausea, absolutely nothing by the mouth, except perhaps a few drops of acidulated ice-cold water to relieve the dryness of the mouth itself, and copious, faintly saline enemata to relieve thirst, though stern treatment from the patient's point of view, is the shortest and best road to relief.—*Med. and Surg. Reporter.*

---

CHLOROFORM DURING SLEEP.—The following case is of interest as bearing on the question whether a sleeping person can be chloroformed without awakening. The reporter was asked to take two teeth out for a girl aged seven, and as she is very timid and excitable, to give her chloroform. On going to her home he found her lying on her back in bed, sound asleep. Having poured about two drachms, probably more, of chloroform on a folded towel, he gradually brought it to about two or three inches from her mouth and held it there. She went on breathing quite quietly, and neither coughing nor making any unwonted movements. In a very short time she was so well under its influence that her hand fell down when raised and the conjunctiva was insensible to touch. She was then lifted out of bed, carried into another room and laid on a sofa without her giving any sign of consciousness. On opening her mouth, however, she put up her hands and turned her head on the pillow. More chloroform was given, and almost immediately she was in a state of complete anæsthesia and the teeth were extracted. She was easily aroused, but almost momentarily fell asleep again and slept for two hours. When she awoke she was much astonished to find that her teeth were out.—*Ther. Gaz.*

---

BYCICLE-RIDING FOR WOMEN.—After pointing out certain contraindications, the *New York Journal of Gynecology and Obstetrics* says, editorially: "But there are a certain number of women in this country, even those long past puberty, who, hard as it is at times to realize it, have never had any pelvic disease whatsoever, and it will be in regard to these that we shall be called upon to answer the question: Is bicycle-riding beneficial for women? We are inclined to think that, under these circumstances, it is. The muscular action is much more regular than in horseback-riding, and the danger of accident, for a good rider, far less. For women past the menopause, with a tendency to excessive flesh, to torpidity of the liver, to constipation and to dyspepsia, it should be of especial benefit. But we fear that in this class of cases, in which it would appear to be most indicated, the power of feminine vanity will ever place a bar to our converting a probable theory into practical experience.

"At all events, there is no other form of exercise within the capacity of the average woman which involves so general a muscular development and glandular action combined with a healthy mental and physical exhilaration."

FOOD AND THE TEETH.—Dr. Stockton, Newark, N. J.—This is a subject that has been variably discussed for a great many years, and I don't know that we are much wiser to day than we were when we first began its consideration. It is said that there is a balm for every wound in the *vis medicatrix* of nature, and if that be true we should be able to discover it, and if it were discovered we certainly should find something that would repair the loss that comes on our teeth ; just as the physician and the scientist of to day are able to discover remedies for other diseases. My attention has been called to a remedy by which, if the gentleman tells the truth, the discoverer is able to cure rheumatism almost invariably. Now, if it is possible that this gentleman has found a remedy for that painful and tormenting disease, why cannot we find a remedy for the ills that beset humanity concerning their teeth ? I am not enough of a scientist to discuss this subject except, in the language of my friend, Dr. Luckey, on general principles; but on these I think there must be something somewhere, that will be found some time, that will place us in a different and a better position from that which we occupy to-day. I know a family in which one boy has splendid teeth, and another and succeeding child in the same family will have teeth as bad as the other's are distinctly good. I know that succeeding children born of the same father by a different mother will have teeth of exactly the same characteristics ; one child will have a most perfect set of teeth, and the other as poor a set as can well be erupted. Why is it ? You would say that the same influences that produce the one perfect and magnificent set of teeth, all things being equal, would produce the same in another child. We know it is not so ; why it is we do not know. I wish I had the vivid imagination and command of language that our friend Dr. Dwinelle had. Before this society, or perhaps some other, he portrayed the influence and effect of animal phosphates on the teeth in contradistinction to mineral phosphate. He told us that in some Eastern country—India or Africa—

the people were dying by thousands of a disease that had formerly yielded to their remedies, though they continued to take, as they supposed, the same remedies that had cured them before; but the man who prepared the medicine had become rich and sold out, and his successor used in the manufacture of the medicine a mineral instead of animal phosphate, and the result of this change was that the people died by thousands. It was finally discovered that they were using a different phosphate; the animal phosphate was substituted, and the people were cured and lived. Now, if we could only discover some remedy of equal certainty for the diseases of the teeth, what a blessing it would be. It is well known that if you feed a dog for a few months on starchy food, his teeth will decay. Therefore there must be something in feeding; and if we knew what it was, we could take into our systems, as is shown in the case of the dog and other animals, something that would remedy this defect.—*International.*

---

TREATMENT OF APPARENT DEATH BY RHYTHMICAL TRACTION UPON THE TONGUE.—The first case in which rhythmical traction of the tongue was employed at Paris to produce artifiicial respiration was by M. Lapicida (*La Tribune Medicale*), aided by a student.

A young woman, wishing to commit suicide, sprang into the Seine. She was seen by two sailors, who went to her rescue; after remaining for five minutes submerged, she was finally withdrawn from the river. Taken at once to the station, frictions were applied, but without success, when M. Lapicida, following the method of Laborde, began rhythmical tractions of the tongue.

At the end of five minutes a slight blowing sound was heard and respirations were established. At the same time the patient experienced a nervous crisis and objected to being resuscitated. She was placed in bed and enveloped in warm clothing. A few moments afterward she vomited freely and all danger was over.

Lapicida, who is chief of the post of the life-saving society, expressed himself as amazed at the rapidity with which the respiratory functions were restored, and resolved to employ this method in all cases which came under his care in the future.

Notices detailing the method of Laborde have been placed in the life-saving stations throughout Paris.—*Med. and Surg. Reporter.*

---

TO SECURE PERFECT ARTICULATION BETWEEN NATURAL AND SUBSTITUTE TEETH IN BRIDGE-WORK.—Take bite in modeling compound, and flow in Melotte's fusible metal for model on which to adjust the teeth. Is very accurate, there is no wearing away of edges as with plaster, and by swaying between metallic casts a very perfect occlusion can be secured. In heating the fusible metal, put a little water in the spoon with the metal, which secures a degree of heat of 512°, and it sets immediately.—*Items of Interest.*

---

TO INCREASE THE DENSITY AND HARDNESS OF AMALGAM FILLINGS.—Heat a portion of the mixed amalgam over a lamp till the mercury volatilizes and the mass becomes comparatively solid. Press this into the soft amalgam already placed into the cavity. Excess of amalgam from previous operations may be used in this way.—*Items of Interest.*

